# Maternal Obesity during Pregnancy Alters Daily Activity and Feeding Cycles, and Hypothalamic Clock Gene Expression in Adult Male Mouse Offspring

**DOI:** 10.3390/ijms20215408

**Published:** 2019-10-30

**Authors:** Jane K. Cleal, Kimberley D. Bruce, Jasmin L. Shearer, Hugh Thomas, Jack Plume, Louise Gregory, James N. Shepard, Kerry L. Spiers-Fitzgerald, Ravi Mani, Rohan M. Lewis, Karen A. Lillycrop, Mark A. Hanson, Christopher D. Byrne, Felino R. Cagampang

**Affiliations:** 1 School of Human Development and Health, Faculty of Medicine, University of Southampton, Southampton General Hospital, Southampton SO16 6YD, UK; j.k.cleal@southampton.ac.uk (J.K.C.); KIMBERLEY.BRUCE@UCDENVER.EDU (K.D.B.); jasminshearer@nhs.net (J.L.S.); hughthomas90@googlemail.com (H.T.); K.L.Hyde@soton.ac.uk (K.L.S.-F.); R.Mani@soton.ac.uk (R.M.); Rohan.Lewis@soton.ac.uk (R.M.L.); M.Hanson@soton.ac.uk (M.A.H.); C.D.Bryne@soton.ac.uk (C.D.B.); 2 Centre for Biological Sciences, Faculty of Natural and Environmental Sciences, University of Southampton, Southampton SO17 1BJ, UK; jp601@exeter.ac.uk (J.P.); Louise.Gregory@soton.ac.uk (L.G.); J.N.Shepard@soton.ac.uk (J.N.S.); K.A.Lillycrop@soton.ac.uk (K.A.L.)

**Keywords:** high fat diet, maternal obesity, pregnancy, circadian clocks, appetite, suprachiasmatic nucleus, arcuate nucleus, mouse, activity

## Abstract

An obesogenic diet adversely affects the endogenous mammalian circadian clock, altering daily activity and metabolism, and resulting in obesity. We investigated whether an obese pregnancy can alter the molecular clock in the offspring hypothalamus, resulting in changes to their activity and feeding rhythms. Female mice were fed a control (C, 7% kcal fat) or high fat diet (HF, 45% kcal fat) before mating and throughout pregnancy. Male offspring were fed the C or HF diet postweaning, resulting in four offspring groups: C/C, C/HF, HF/C, and HF/HF. Daily activity and food intake were monitored, and at 15 weeks of age were killed at six time-points over 24 h. The clock genes *Clock*, *Bmal1*, *Per2*, and *Cry2* in the suprachiasmatic nucleus (SCN) and appetite genes *Npy* and *Pomc* in the arcuate nucleus (ARC) were measured. Daily activity and feeding cycles in the HF/C, C/HF, and HF/HF offspring were altered, with increased feeding bouts and activity during the day and increased food intake but reduced activity at night. Gene expression patterns and levels of *Clock, Bmal1*, *Per2*, and *Cry2* in the SCN and *Npy* and *Pomc* in the ARC were altered in HF diet-exposed offspring. The altered expression of hypothalamic molecular clock components and appetite genes, together with changes in activity and feeding rhythms, could be contributing to offspring obesity.

## 1. Introduction

There is an escalating global epidemic of obesity that has deleterious consequences on the health of the population, with crippling effects on the global economy [1]. Obesity is a consequence of changes in nutritional homeostasis, which is a basic biological process to balance food intake with energy expenditure. Thus, the consumption of high amounts of energy, particularly fat and sugar, which are not burned off through increased physical activity and exercise leads to obesity. In humans, obesity is defined as having a body mass index (BMI) greater than 30 kg/m^2^ or higher [2]. In rodents, there is no defined threshold for obesity based on body mass index (BMI), and because of a lack of information on anthropometrical parameters, obesity is usually taken as any significant increase in body weight and percent body fat. This definition relies on the assumption that the control animals maintained in the laboratory are both lean and normal.

One of the key components in nutritional homeostasis is the maintenance of daily rhythms in sleep–wake cycle, locomotor activity, feeding behavior, and energy metabolism across the 24 h (circadian) light/dark cycle, which is actively coordinated by a central circadian clock located in the suprachiasmatic nucleus (SCN) of the hypothalamus [3]. The molecular basis of circadian oscillations is composed of positive and negative transcriptional–translational feedback loops [4,5,6]. The process starts with the dimerisation of the CLOCK and BMAL1 proteins that bind to a specific E-box regulatory element in the promoter of the clock genes *Period* (*Per*) and *Cryptochrome* (*Cry*) to induce transcription. These transcripts then produce proteins that form dimers after translation to repress their transcription by competing with CLOCK/BMAL1 binding. Following the degradation of the inhibitory proteins, this transcription–translation feedback loop starts over for another circadian cycle. The link between the molecular circadian clock in the SCN and metabolism has been elucidated by well-documented studies in *Clock*-deficient mice, which exhibited obesity and hyperphagia [7,8]. More recent studies have shown that an obesogenic high fat (HF) diet leads to disruption in circadian locomotor activity, food intake, and hepatic clock gene expression [9,10].

The arcuate nucleus (ARC) in the hypothalamus plays an important role in regulating energy balance via the central melanocortin system [11]. The melanocortin system is composed of various types of neurons that produce pro-opiomelanocortin (POMC) and neuropeptide Y (NPY) [12]. The POMC and NPY neurons represent two main neuronal populations with antagonistic functions in the regulation of energy intake and expenditure, where on one hand stimulation of POMC neurons inhibits food intake [13,14], the stimulation of NPY neurons by exogenous NPY administration promotes food intake [15,16,17]. Alterations in hypothalamic mRNA levels of *Npy* and *Pomc* are associated with obesity [18,19,20,21,22]. Studies have revealed the importance of circadian integration by the SCN of ARC function driving the daily changes in levels of these transcripts to regulate energy metabolism [23,24]. The SCN communicates with the ARC to generate circadian rhythm in feeding behaviour [25]. In addition to receiving SCN projections, the ARC also has autonomous circadian oscillators [26]. Likewise, the link between the SCN and the ARC is essential to maintain the endogenous rhythmic locomotor activity [27].

One of the consequences of the increasing rate of the current obesity epidemic in the general population is an increased prevalence of obesity affecting one in five pregnancies. Obesity during pregnancy leads to immediate and long-term complications for maternal and fetal health. Obese women are more likely to develop pregnancy disorders such as pre-eclampsia and gestational diabetes, while offspring born to obese mothers have an increased risk of developing metabolic diseases, such as type 2 diabetes and obesity in later life through a process known as developmental programming [28]. In non-human primates, maternal consumption of an obesogenic HF diet during pregnancy alters clock gene expression in the fetal and juvenile livers [29]. We and others have reported in rodents that hepatic mRNA expression of clock genes is profoundly altered in offspring exposed to maternal obesity, contributing to impaired metabolism in the offspring livers [30,31]. However, the ability of maternal obesity and/or exposure to HF diets during early development to directly alter hypothalamic clock gene function has yet to be determined.

We therefore tested the hypothesis that diet-induced obesity during pregnancy in mice could contribute to the development of offspring obesity by altering the daily expression patterns of molecular clock components and appetite genes in the offspring brain and disrupting their activity and feeding behaviour. Our aim was to track food consumption and activity of the offspring over 24 h under a 12 h light–12 h dark (LD) cycle. Offspring were analysed every 4 h across the 24 h LD period and transcript levels of clock genes and the genes that regulate appetite were measured in the SCN and ARC, respectively.

## 2. Results

### 2.1. Maternal Obesity and High-Fat Diet Alters Metabolic Phenotype and Behaviour in the Offspring

The weights of the dams fed the HF diet were significantly heavier at the time of conception vs. those on control diet (37.6 ± 0.9 g vs. 27.3 ± 0.8 g, respectively; *p* < 0.001). These HF-fed dams have greater amount of body fat compared to those on the control diet (7.5 ± 1% BW vs. 22.5 ± 2% BW, respectively; *p* < 0.001). In the 15-week-old male offspring, we measured the body weight and total body fat, as well as the amount of food and activity during the day and at night to evaluate if maternal obesity and/or postweaning feeding of a HF diet altered their metabolic phenotype and feeding behaviour. Offspring from obese HF-diet-fed dams that were fed from postweaning with the control (C) diet (i.e., the HF/C offspring) were significantly heavier than the C/C offspring (*p* < 0.05), but in turn lighter (*p* < 0.05) than offspring of lean C-fed dams fed a HF diet postweaning (C/HF offspring) (Table 1). Offspring of obese dams that were also fed from postweaning with a HF diet (HF/HF offspring) was the heaviest among the four offspring group (*p* < 0.05). Two-way ANOVA further revealed that the variance seen between the groups was attributed to both the effect of maternal obesity and postweaning HF diet consumption (both at *p* < 0.0001) but with no interaction between maternal obesity and postweaning feeding of the HF diet (Table 1). These significant differences in body weight were reflected in terms of the offspring’s percentage body fat (Table 1). Two-way ANOVA attributed the variance in adiposity to both maternal obesity and postweaning HF diet consumption (both at *p* < 0.0001) with no interaction between the variables as responsible for the variance.

**Figure 1:**
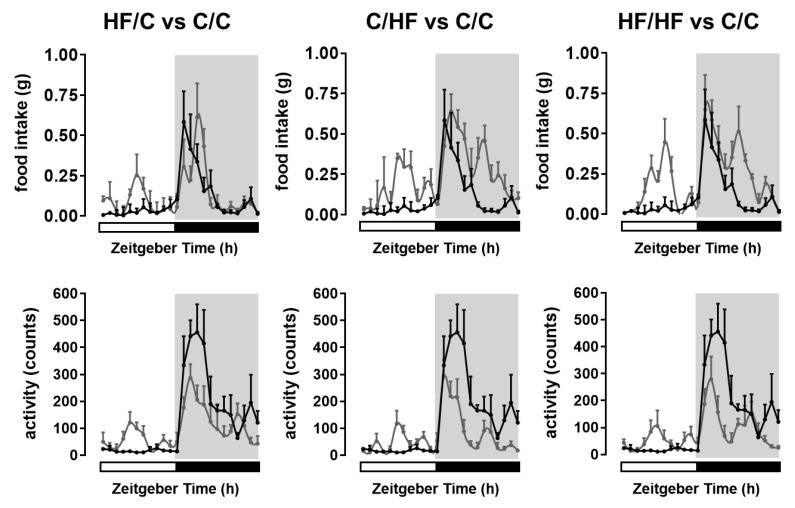
Maternal obesity and postweaning high-fat (HF) diet consumption alters diurnal rhythms of food intake and activity. The 24 h food intake and activity during the light–dark cycle were recorded for the offspring from lean dams and on the control (C) diet (C/C offspring, black lines) and compared with the control-fed offspring from obese dams (HF/C offspring, grey lines), or with the HF-fed offspring from lean (C/HF group) or obese (HF/HF group) dams (grey lines).

In the case of feeding pattern, there was a significant increase (*p* < 0.05) in daytime feeding in the offspring fed the HF diet postweaning and/or from obese dams (i.e., the HF/C, C/HF, and HF/HF offspring) (Figure 1 and Table 1). Two-way ANOVA indicates that the variance in daytime feeding was attributable to both maternal obesity and postweaning HF diet consumption (both at *p* < 0.0001), but also there was a significant interaction between these two variables on the overall variance (*p* < 0.0001). At night, there was a significant increase in feeding (*p* < 0.05) as a consequence of postweaning HF diet consumption in the C/HF and HF/HF offspring (Table 1) but not in the HF/C offspring. Two-way ANOVA indicates that the variance in nighttime feeding was attributed to both maternal obesity and postweaning HF diet consumption (both at *p* < 0.0001), but there was also a significant interaction between these two variables on the overall variance (*p* < 0.0001) (Table 1). Thus, maternal HF diet feeding seems to trigger only a daytime increase in food intake in the HF/C offspring. By contrast, postweaning HF diet feeding with or without maternal obesity (i.e., in HF/HF and C/HF offspring, respectively) appears to induce an increase food intake not only during the daytime but also during the latter part of the night (Figure 1).

In the case of locomotor activity pattern, there were significant increases (*p* < 0.05) in daytime activity in the HF/C, C/HF, and HF/HF offspring (Figure 1 and Table 1). Two-way ANOVA indicates that the variance in daytime activity was attributable to both maternal obesity and postweaning HF diet consumption (both at *p* < 0.0001), but also there was a significant interaction between these two variables on the overall variance (*p* < 0.0001). There were significant reductions (*p* < 0.05) in nighttime activity in the HF/C, C/HF, and HF/HF offspring (Figure 1 and Table 1). Two-way ANOVA revealed that both maternal obesity and postweaning HF diet consumption contributed to the variance (both at *p* < 0.0001), but also there was a significant interaction between these two variables on the overall variance (*p* < 0.0001) (Table 1). Overall, HF-diet-induced maternal obesity seems to trigger alteration in locomotor activity patterns regardless of the postweaning feeding condition imposed on the offspring (Figure 1).

### 2.2. Maternal Obesity and High-Fat Diet Disrupts the Daily Expression Patterns of Clock Gene in the Suprachiasmatic Nucleus (SCN) of the Offspring Brain

We examined the mRNA expression of the clock gene components in the SCN to determine if maternal obesity and/or exposure to postweaning HF diet altered circadian rhythms over a 24 h LD period. The results are presented as ANOVA and cosinor analysis of the clock genes *Clock*, *Bmal1*, *Per2*, and *Cry2* in the SCN of offspring over six separate time points. 

**Figure 2:**
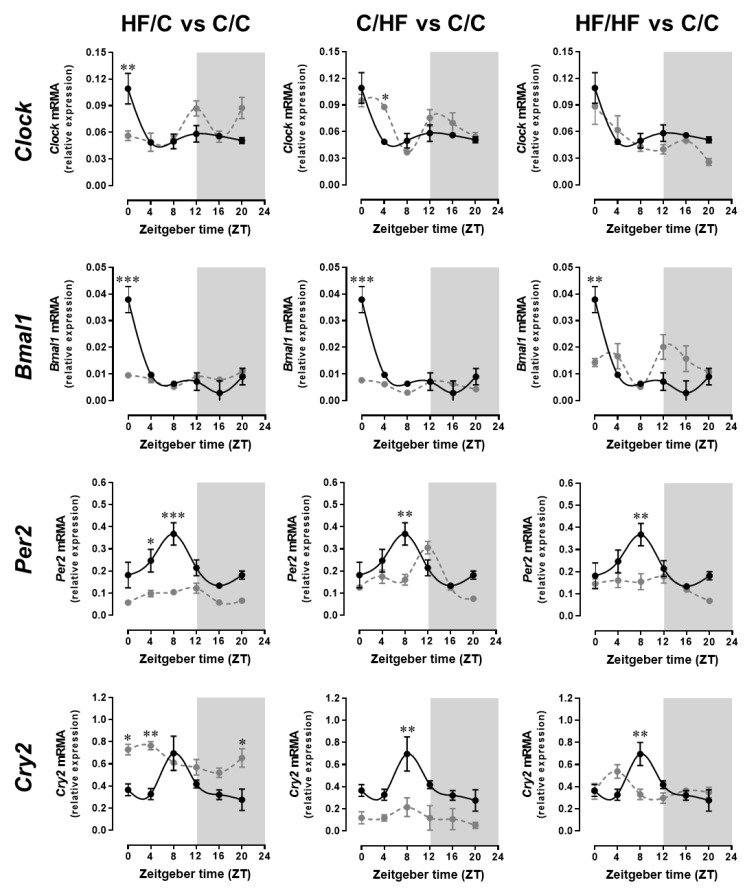
Maternal obesity and postweaning HF diet consumption disrupts circadian rhythms of clock gene expression in the suprachiasmatic nucleus (SCN). Transcripts of the core clock genes *Clock*, *Bmal1*, *Per2*, and *Cry2* in the SCN were analysed by real-time PCR. Tissues were harvested every 4 h from 15-week-old male offspring from lean dams and on the control diet (C/C offspring, black lines), control-fed offspring from obese dams (HF/C offspring, grey lines), or with the HF-fed offspring from lean (C/HF group) or obese (HF/HF group) dams (grey lines). Values are displayed as relative expression (mean ± SEM) after normalisation to the housekeeping genes *β-actin* and *Gapdh*. ANOVA with Tukey post hoc test. * *p* < 0.05, ** *p* < 0.01, *** *p* < 0.001 between groups at each time point.

The positive arm of the transcriptional–translational feedback loop of the central clock machinery involves BMAL1 which dimerises with CLOCK to form a heterodimer that binds with E-box promoter elements to induce the transcription of the other clock components including *Cry* and *Per*, as well as other clock-controlled genes. We observed that the mRNA expression levels of *Clock* and *Bmal1* were elevated during the day and were low at night in the SCN of offspring on control diet and from lean dams fed the same control diet (C/C offspring) (Figure 2). ANOVA analysis shows significant difference in *Clock* gene expression between the C/C offspring vs. the HF/C offspring at ZT0 (*p* < 0.01) and vs. the C/HF offspring at ZT4 (*p* < 0.05). Two-way ANOVA revealed that maternal obesity had the main effect on the observed variance (*p* < 0.001), but there was also a significant interaction of maternal obesity and postweaning HF diet consumption (*p* < 0.001) on the overall variance. ANOVA analysis of *Bmal1* gene expression showed significant difference between the C/C offspring vs. the HF/C and C/HF offspring (both at *p* < 0.001) and the HF/HF offspring (*p* < 0.01) at ZT0. Two-way ANOVA revealed that maternal obesity and postweaning HF diet both contribute to the variance (both at *p* < 0.05), but also there was a significant interaction between these variables (*p* < 0.001) on the overall variance. 

The negative arm of the transcriptional–translational feedback loop that impairs the action of the CLOCK/BMAL1 to complete this loop includes *Cry* and *Per*. The mRNA expression levels of *Per2* and *Cry2* in the SCN were elevated during the latter part of the day (ZT8) and low at night in offspring on the control diet and from control diet-fed lean dams (C/C offspring) (Figure 2). ANOVA analysis showed significant difference in *Per2* gene expression between the C/C offspring vs. the HF/C offspring at ZT4 (*p* < 0.05) and at ZT8 (*p* < 0.001) and vs. the C/HF and the HF/HF offspring both at ZT8 (*p* < 0.01). Two-way ANOVA revealed that maternal obesity had the main effect on the observed variance (*p* < 0.01), but there was also a significant interaction of maternal obesity and postweaning HF diet consumption (*p* < 0.05) on the overall variance. ANOVA analysis of *Cry2* gene expression showed significant difference between the C/C offspring vs. the HF/C offspring at ZT0 and ZT20 (both at *p* < 0.05) and at ZT4 (*p* < 0.01), vs. the C/HF and the HF/HF offspring both at ZT8 (*p* < 0.01). Two-way ANOVA revealed that this was attributed to both maternal obesity and postweaning HF diet consumption (*p* < 0.001) but with no interaction between these variables.

Cosinor analysis revealed rhythmicity in *Clock* in the SCN of the C/C offspring (*p* < 0.05) (Table 2) with peak expression at 23.41 h. Rhythmicity in *Clock* was also detected in the HF/HF offspring but with a phase shift of 2.87 h (*p* < 0.05). No significant *Clock* gene rhythmicity was found in both the C/HF and the HF/C offspring. The adjusted mean (mensor) yielded no significant differences in *Clock* between the offspring groups. Cosinor analysis also showed rhythmicity in *Bmal1* in the SCN of the C/C offspring (*p* < 0.05) peaking at 0.39 h. The adjusted mean revealed significant reduction (*p* < 0.05) in *Bmal1* in the C/HF and HF/C offspring groups, but no significant *Bmal1* rhythmicity was detected in the C/HF, HF/C, and HF/HF offspring. Cosinor analysis yielded rhythmicity in *Per2* in the SCN of C/C offspring (*p* < 0.05) with peak levels at 7.01 h. There was a phase shift of 3 h (*p* < 0.05) in the rhythmicity found in the HF/C offspring. No significant *Per2* rhythmicity was detected in the C/HF and HF/HF offspring groups, but there was a significant reduction in the adjusted mean (*p* < 0.05) in the C/HF offspring. Rhythmicity in *Cry2* was detected by cosinor analysis in both C/C and HF/C offspring (*p* < 0.05). There was a phase shift of 6.33 h (*p* < 0.05) and a significant reduction in the adjusted mean (*p* < 0.05) in the HF/C offspring.

### 2.3. Maternal Obesity and High-Fat Diet Alters the Daily Expression Patterns of Appetite Genes in the Arcuate Nucleus (ARC) of the Offspring Brain

**Figure 3:**
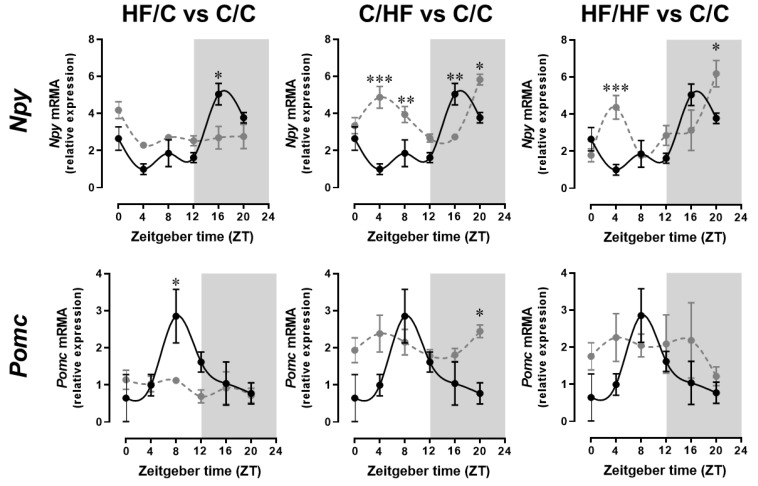
Maternal obesity and postweaning HF diet consumption alters rhythmic pattern of appetite gene expression in the arcuate nucleus (ARC). Transcripts of the genes that regulate food intake *Npy* and *Pomc* in the ARC were analysed by real-time PCR. Tissues were harvested every 4 h from 15-week-old male offspring from lean dams and on the control diet (C/C offspring, black lines), control-fed offspring from obese dams (HF/C offspring, grey lines), or with the HF-fed offspring from lean (C/HF group) or obese (HF/HF group) dams (grey lines). Values are displayed as relative expression (mean ± SEM) after normalisation to the housekeeping genes *β-actin* and *Gapdh*. ANOVA with Tukey post hoc test. * *p* < 0.05, ** *p* < 0.01, *** *p* < 0.001 between groups at each time point.

We also examined the transcript levels of the genes that regulate food intake (*Npy* and *Pomc*) in the ARC to determine maternal obesity and/or exposure to the postweaning HF diet altered their expression patterns over a 24 h LD period (Figure 3). The results are presented into ANOVA and cosinor analysis of the appetite gene *Npy* and *Pomc* in the ARC of offspring over six separate time points. 

ANOVA analysis show significant difference in *Npy* gene expression between the C/C offspring vs. the HF/C offspring at ZT16 (*p* < 0.05), vs. the C/HF offspring at ZT4 (*p* < 0.001), ZT8 and ZT16 (both at *p* < 0.01), and at ZT20 (*p* < 0.05, and vs. the HF/HF offspring at ZT4 (*p* < 0.001) and at ZT20 (*p* < 0.05). Two-way ANOVA revealed that postweaning HF diet consumption had the main effect on the observed variance (*p* < 0.01), but there was also a significant interaction of maternal obesity and postweaning HF diet consumption (*p* < 0.01) on the overall variance. ANOVA analysis of *Pomc* gene expression showed significant difference between the C/C offspring vs. the HF/C offspring at ZT8 (*p* < 0.05), and vs. the HF/HF offspring at ZT20 (*p* < 0.05). Two-way ANOVA revealed that postweaning HF diet consumption had the main effect on the variance (*p* < 0.05) but with no interaction between maternal obesity and postweaning HF diet consumption.

Cosinor analysis revealed rhythmicity in *Npy* in the ARC of the C/C offspring (*p* < 0.05) (Table 2) with peak expression at 17.97 h. No significant *Npy* gene rhythmicity was found in the HF/C, C/HF, and HF/HF offspring. Cosinor analysis also showed rhythmicity in *Pomc* in the ARC of the C/C offspring (*p* < 0.05), peaking at 9.27 h. There was also no significant *Pomc* gene rhythmicity in the HF/C, C/HF, and HF/HF offspring, and the adjusted mean revealed significant increase (*p* < 0.05) in *Pomc* in the C/HF offspring.

## 3. Discussion

Evidence from a growing number of clinical and animal studies demonstrates that obesity and other metabolic diseases may have developmental origins. Our findings suggest diet-induced maternal obesity during pregnancy increases offspring susceptibility to becoming obese and these phenotypic changes are accompanied by altered daily expression patterns of molecular clock components in the SCN and genes involved in appetite regulation in the ARC in the offspring hypothalamus. These molecular changes coupled with the observed alterations in the daily activity cycle and feeding patterns could increase the likelihood of the offspring developing obesity in adulthood.

The increased body weight and adiposity in the offspring of obese dams observed in this study recapitulate our previous findings using the same maternal feeding protocol and diets [32,33,34] and are comparable with results from other studies done in rodents showing diet-induced maternal obesity increasing both offspring body weight and adiposity [35,36]. In this study, we aimed to understand the possible explanations as to why these offspring are more susceptible to becoming obese. Here we show that the HF diet-induced maternal obesity alters offspring feeding behaviour. Whilst control-fed offspring from lean dams (C/C group) consumed the majority of their food during the dark period of the 12 h light–12 h dark cycle, which is the typical feeding behaviour for this mouse strain [37,38], the offspring of obese dams showed increased bouts of feeding during the day resulting in the overall increase in their daily food intake. Although other studies have previously reported in rodents of increased food intake in offspring from obese dams [35] or in offspring of lean dams fed diets high in nutritional fat [39,40], none of these studies have characterised the timing of their feeding bouts and have simultaneously tracked their activity. Our study shows for the first time that increased daily food intake is due to increased feeding activity both during the day and at night. The increase in both daytime and nighttime feeding bouts was more pronounced when the offspring were on the HF diet irrespective of whether the dams were obese or lean. The increased activity, albeit to a lower level during the day, in the offspring of obese dams fed either the HF diet or control diet as well as in the control-fed offspring from obese dams corresponds to the period when these offspring are actively feeding. However, nighttime activity levels were significantly lower in all three offspring groups in spite of increased feeding bouts. Thus, maternal obesity seems to trigger alteration in locomotor activity patterns whatever postweaning feeding condition is imposed on the offspring. Taken together, the increase in daily energy intake and reduced energy expenditure at night could be contributing to the offspring becoming obese. 

A similar observation of a significant reduction in nighttime activity measured by radio-telemetry was reported in three-months-old male mouse offspring from obese dams [36] but the study did not observe increased activity during the day. In the present study, activity during the day was associated with the timing of feeding bouts during this period. This difference in daytime activity between the two studies could be due to different systems used to measure activity or age difference when the activity was monitored in the offspring. The previous study measured activity in three-month-old male offspring while we measured the offspring at a younger age (about 2 months old). This would suggest that the changes in food intake and activity continue to evolve during the various phases in the offspring growth trajectory. This notion is substantiated by observations made in the same previous study reporting that the reduction in nighttime activity was not observed anymore at six months of age in the male offspring from obese dams [36].

We also investigated central neural circuits that regulate activity and feeding behaviours, focusing on the circadian clock located in the SCN and neurons important in the regulation of appetite in the ARC, to determine whether maternal obesity during pregnancy and postweaning HF diet consumption alter gene expression patterns in these hypothalamic nuclei. The observed peak expressions of *Bmal1*, *Per2,* and *Cry2* in the C/C offspring SCN were similar to what was found in previous studies [41,42,43,44]. However, others have observed constitutive gene expression of *Clock* in the SCN [45] while in the present study we observed rhythmic expression of *Clock* in the C/C offspring SCN that peaks just before light onset. The conflicting observation in Clock gene expression could be due to differences in the method used to take out the SCN. Whilst we micropunched the SCN, others have taken hypothalamic slices which could be contaminated by extra-SCN areas of the hypothalamus. In the present study, we also show that peak *Npy* mRNA levels in the ARC were found during the early subjective night in the C/C offspring. This is in contrast to previous observation in rats kept in LD condition where peak *Npy* levels were detected during the day prior to dark onset [46] or just after light onset [23]. It is not clear from these studies whether the rats were killed during the dark phase under dim red light, but we used night vision goggles when we were sampling our mice during this period in the LD cycle. We also measured *Npy* mRNA levels by PCR while previous studies used in situ hybridisation and a computerised image analysis system to measure cellular levels of *Npy* mRNA. As for the gene expression profile of *Pomc* in the ARC of the C/C offspring, we observed similar peak levels during the day to those found in previous studies in rats kept in LD condition [23,47].

The maintenance of circadian rhythms in the SCN plays an important role in optimising the integration of neural circuits regulating food intake and activity. Alterations in diet composition (high-fat diets), the timing of eating, or lifestyle factors (sleep deprivation or night-shift work) are known to alter circadian rhythms and impair metabolism resulting in obesity [48,49,50,51,52,53,54]. Here, we show alterations in the daily rhythms and transcript levels of both *Per2* and *Cry2* in the SCN of offspring from obese dams. Since *Per2* and *Cry2* dimerise and inhibit *Clock* and *Bmal1* [6], the overall function of the central clock system that maintains circadian rhythms is impaired and this, in turn, could compromise the integration of central neural circuits regulating food intake and activity. This could explain how maternal obesity and HF diet-induced circadian clock gene disturbance results in altered circadian activity patterns. Whilst we studied SCN tissue for gene-expression changes responsible for the altered 24-h activity, multiple brain areas are known to influence activity. The circadian clock system is organised hierarchically with the SCN synchronizing the clocks in other brain areas and peripheral tissues. The SCN drives daily activity rhythms by secreting factors including transforming-growth-factor-alpha [55] which act on secondary areas within the medial and lateral hypothalamus to stimulate the reticulospinal tract to initiate voluntary movement [56]. The reticulospinal tract also integrates information from nuclei of the basal ganglia [56], thus we cannot attribute the observed changes in activity rhythms entirely to changes in SCN gene-expression as downstream locomotor activity will be a result of multiple clock-modified signals. This notion is substantiated by our observation that daily locomotor activity pattern is still apparent in the offspring with modified diets and/or from obese dams albeit the patterns having been altered even though we find that the expression rhythms of the clock genes investigated are abolished in these offspring. As we did not measure the expression patterns of the other clock and clock-controlled genes, including *Per1*, *Cry1,* and *Rev-erb alpha*, it is therefore possible that the rhythmic expressions of these genes are not abolished to the same extent by the HF diet consumption and/or maternal obesity thus maintaining the daily locomotor activity pattern in these offspring. 

The alteration in feeding activity in the offspring of obese dams was accompanied by observed changes in both levels and daily patterns of *Npy* and *Pomc* expression in the hypothalamic ARC. The stimulation of POMC neurons in the ARC inhibits food intake [13,14] while the activation of NPY neurons in the ARC leads to increase food consumption [15,16,17]. Studies have revealed the importance of circadian integration by the SCN of ARC function driving the daily changes in levels of *Npy* and *Pomc* to regulate feeding behaviour [23,24]. Thus, the increased feeding activity during the dark period in these nocturnal mice could be linked to the observed nighttime increases in *Npy* expression levels in the ARC, while the elevated *Pomc* transcript levels in the ARC could be inhibiting feeding drive during the day. It is possible that the daytime increase in ARC *Npy* mRNA levels coupled with altered expression patterns in daytime *Pomc* levels in HF-fed offspring from lean and obese dams, as well as in control-fed offspring from obese dams, is driving the daytime feeding bouts that we have observed in these offspring. The elevated expression of *Pomc* in the ARC in the HF-fed offspring from either lean or obese dams could be due to the increase in circulating glucose due to the HF diet, and this is influencing the expression of this gene in the ARC. Recent evidence has shown that there are glucose-responsive POMC-containing neurons in the hypothalamus that increase in activity in response to extracellular glucose [57]. This suggests that the increased *Pomc* in the ARC in the HF-fed offspring from either lean or obese dams could result in reduced feeding activity at night. Conversely, it has been reported that chronic feeding of the HF diet significantly reduced the number of hypothalamic POMC neurons [58] and the loss of these cells is sufficient to increase feeding and cause excess weight gain [59]. It would therefore be interesting to find out if the consumption of the HF diet during pregnancy results in a reduction of POMC neurons in the hypothalamic ARC. Several studies have also shown that central administration of fatty acids can increase hypothalamic NPY mRNA levels [60] and that the action of NPY neurons can also directly inhibit POMC neurons in the ARC [61]. Hence, the observed elevation in *Npy* levels in the ARC during the day and at night could reflect an increase in NPY neuronal activity in the offspring fed the HF diet. This, coupled with a possible reduction in the number of POMC neurons, could result in the increased feeding bouts in these offspring. 

Although we found that changes in mRNA levels of *Npy* and *Pomc* in the ARC are due mainly to the postweaning HF diet, a previous study in rats has shown that maternal obesity during pregnancy can already alter mRNA expression of *Pomc* and *Npy* in the fetal brain ARC [62]. It has been suggested that this facilitates an earlier onset of independent feeding [63], which may explain the increased food intake and altered feeding behaviour that we observed in control-fed offspring from obese dams. However, *Npy* and *Pomc* are not exclusively controlled by gene expression. Hormonal factors including insulin, leptin, and ghrelin, as well as circulating metabolites such as glucose and fatty acids also influence the activity of NPY and POMC neurons [24,58,60]. Likewise, NPY and POMC are not only active in the ARC as they have projections to several other hypothalamic nuclei [14]. Appetite is a psychological desire for food, involving brain areas implicated in pleasure and reward, specifically the nucleus accumbens, ventral tegmental area, prefrontal cortex, amygdala, and septum [64]. Furthermore, other factors besides appetite influence food intake. Hunger is a physiological requirement for food, either calories or specific nutrients [65,66], while satiety is the sensation of fullness [66], generated by various factors including gut hormone secretions following a large meal, vagal afferent stimulation by stomach distension, insulin and glucagon secretion, and blood glucose concentration [14]. These systems may also be affected by maternal obesity and the consequent changes in clock gene function in the offspring brain. Indeed, studies in rats have shown that feeding the dams a high-fat, high-sugar diet results in altered development of the reward system in the offspring brain, resulting in increased fat intake and alterations in the response of the reward system to excessive caloric intake in postnatal life [67].

Although we used the term ‘HF diet’, it is not just the amount of fat in the diet that is different compared to the control diet but also the amount of carbohydrate. Furthermore, the control diet has the equivalent amounts of monounsaturated and polyunsaturated fatty acids (MUFA and PUFA, respectively), while the saturated fatty acid (SFA) content was about half of each of these. The HF diet, on the other hand, contained similar amounts of SFA and MUFA, while the PUFA content was about two-thirds of each of these. It has been reported previously that classes of fatty acids can alter the structure and function of the hypothalamus [68,69]. It is possible that the effects we are reporting here could be explained by the differences in the lipid composition of the diets and not necessarily by the obese condition. The study was also conducted using male offspring and there may be sex differences in the effect of maternal obesity and the HF diet on molecular clock components and appetite genes, as well as activity and feeding rhythms. In a previous study, subtle sex differences in the circadian rhythms of activity, neuronal physiology, and gene expression were observed in mice on laboratory chow diet under a 12 h light–12 h dark (LD) cycle [70]. It would therefore be interesting to examine if maternal obesity and postweaning HF feeding result in differences in the expression patterns of the clock and appetite genes, as well as activity and feeding rhythms between male and female offspring. However, our previous study has shown similar patterns in weight gain and increased adiposity between HF-fed male and female offspring of lean and obese dams at 15 and 30 weeks old [32]. The offspring in the present study were kept under LD condition, hence the endogenous rhythms generated are synchronised by the day and night cycle. It is possible that alterations in the diurnal expression patterns of the clock and appetite genes, as well as activity and feeding rhythms, change differently in the absence of the LD stimuli. Thus, to assess the robustness of circadian rhythmicity the offspring could be transferred to constant darkness to evaluate their free running rhythms. We also measure mRNA levels of the clock and appetite genes in the SCN and ARC, respectively, and it is possible that the translational process could also be affected by maternal obesity and postweaning HF diet consumption resulting in different expression patterns and levels of clock and appetite proteins in these brain loci. Epigenetic mechanisms have also previously linked maternal HF diet to obesity in the offspring [71,72]. Studies in rats have shown that maternal HF diet feeding can result in epigenetic changes in the regulatory regions of *Pomc* in the rat offspring hypothalamus [73]. Obesity has also been associated with DNA methylation status of the clock genes *Clock*, *Bmal1*, and *Per2* [74]. In mice, demethylation in the E-box region of the *Per1* promoter specific to the SCN has been observed during the perinatal period [75] and *Clock* itself has intrinsic histone acetyltransferase activity [76]. Thus, altered expression rhythms of clock genes in the SCN and the appetite genes in the ARC of the offspring could be due to epigenetic changes brought about by maternal obesity.

In conclusion, we have shown that the HF diet-induced maternal obesity during pregnancy alters the daily expression rhythms of clock genes in the SCN and appetite genes in the ARC of the offspring brain. This was accompanied by observed changes in the offspring locomotor activity and feeding behaviour. Given that the rate of obesity in women of child-bearing age is increasing worldwide [77] and the offspring of these obese mothers are predisposed to being overweight or obese [78], the observed gene expression changes in the offspring brain coupled with alterations in locomotor and feeding behaviour, could be some of the factors affecting the increasing obesity in future generations.

## 4. Materials and Methods 

### 4.1. Animals

All animal procedures were carried out at the University of Southampton, in accordance with the regulations of the United Kingdom Animals (Scientific Procedures) Act 1986 and were conducted under Home Office Project Licence number 70/6457. The study received institutional approval from the University of Southampton Biomedical Research Facility Research Ethics Committee. Female C57/BL6J mice were housed under a 12 h light–12 h dark (LD) cycle (lights on at 07:00), and at a constant temperature of 22 ± 2 °C with food and water available ad libitum. These females were randomly assigned to one of two diets: control (C; 7.4% kcal fat; RM1-SDS diet; Special Dietary Services UK), or a high-fat diet (HF; 45% kcal fat; SDS 824053; Special Dietary Services UK) (for more detailed dietary constituents, see Table A1 from Appendix A). We have previously used this HF diet to induce an obese phenotype in both the pregnant dams and their offspring [31,79,80]. The animals were fed their designated diet 8 weeks pre-pregnancy, through to pregnancy and lactation. Pregnant dams were allowed to deliver their pups, and litter size was standardised to six pups to ensure that no litter was nutritionally biased. The male offspring were randomly assigned to either the C or HF diet at weaning at 3 weeks of age resulting in four offspring groups; C/C, C/HF, HF/C, and HF/HF. These offspring were fed their assigned diets for the next 12 weeks. 

### 4.2. Behavioural Analysis

At 15 weeks of age, a subset of mice from the four offspring groups (C/C, C/HF, HF/C, and HF/HF; *n* = 5–6 per offspring group) were randomly selected and individually placed in a physiological cage system with an extensiometric weight transducer that continuously measure food intake and activity (Panlab SLU, Spain). After a 24 h period of acclimatisation, the food intake and locomotor activity were recorded over the next 24-h LD cycle. Subsequently, subsets of mice (*n* = 5–6 per time point per offspring group) were killed by cervical dislocation at six time points during the LD period beginning at 07:00 (designated as ZT0 and defined as the transition time from dark to light period of the LD cycle), then at 11:00 (ZT4), 15:00 (ZT 8), 19:00 (ZT12), 23:00 (ZT16), and 03:00 (ZT20). Animals were killed in complete darkness during the dark phase of the sampling period (ZT12, ZT16, and ZT20) with the aid of head-mounted night vision goggles (Yukon Advance Optics, Thomas Jack’s Ltd., UK). The brains were immediately dissected, frozen in dry ice and stored at −80 °C.

### 4.3. Collection of SCN and ARC Samples

The bilateral suprachiasmatic nuclei (SCN) and arcuate nuclei (ARC) nuclei were dissected out from frozen coronal sections using a Palkovits punch technique [81]. Briefly, frozen brains were cut on a cryostat and 300-µm-thick coronal sections containing the SCN and the ARC were obtained and placed on prechilled glass slides. The bilateral SCN and ARC from adjoining coronal sections were punched out using a brain punch (1.25 mm size, Stoelting Europe, IRL) and stored separately at −80 °C. The sections were then fixed and stained with cresyl violet to check for the accuracy of the SCN and ARC punches.

### 4.4. RNA Extraction and Quantitative Real-Time PCR

Total RNA was isolated from the micropunched SCN and ARC samples using Trifast reagent (Peqlab, Germany) and reverse-transcribed using reverse M-MLV transcriptase (Promega, UK). RNA expression was determined by real-time PCR analysis performed as described previously using an Applied Biosystems 7500 Fast Real-Time PCR System (Thermo Fisher Scientific, UK) [31] to measure the relative amounts of the clock genes (*Clock*, *Bmal1*, *Cry2*, *Per2*) in the SCN samples and the appetite-regulating genes *Pomc* and *Npy* in the ARC samples. We used the housekeeping gene (HKG) *β-actin* and *Gapdh* to normalise the expression levels since we have established that these were the most stably expressed genes following challenge with HF diets and at different circadian time points [82].

### 4.5. Statistical Analysis

Data are expressed as means ± standard error of the mean (SEM). The difference in all measured parameters between 15-week-old male offspring of lean and obese dams fed the control or the HF diet was analysed using one-way and two-way ANOVA, as indicated. Statistical significance was assumed as *p* < 0.05. Cosinor analysis was employed, in addition to ANOVA, to determine rhythmicity of circadian clock gene and appetite gene expression within a 24-h period. Cosinor analysis evaluates the ‘mesor’ (circadian rhythm adjusted mean, based on the parameters of a cosine function), timing of the oscillatory crest, and amplitude, with *p* < 0.05 regarded as significant. Two-way ANOVA was also used to determine the effect of maternal obesity and postweaning HF diet consumption on the observed variance. Statistical analysis was performed using Prism 7.0 software (GraphPad Software, San Diego, CA, USA).

## Figures and Tables

**Table 1:** Phenotypic and behavioural profiles of the offspring.

	Offspring of Lean C-fed Dams		Offspring of Obese HF-fed Dams		*p*-Values		
					Maternal Obesity × Postweaning Diet	Effect of Maternal Obesity	Effect of Postweaning HF Diet
	Control (C/C)	HF Diet (C/HF)	Control (HF/C)	HF Diet (HF/HF)			
Body weight (g)	24.2 ± 0.7 ^a^	33.4 ± 0.4 ^b^	30.6 ± 0.7 ^c^	41.5 ± 0.8 ^d^	ns	<0.0001	<0.0001
Total body fat (%BW)	5.2 ± 0.5 ^a^	12.8 ± 0.9 ^b^	9.2 ± 1.2 ^c^	17.5 ± 1.5 ^d^	ns	<0.0001	<0.0001
Daily food intake (g)							
Daytime	0.40 ± 0.01 ^a^	1.82 ± 0.03 ^b^	1.03 ± 0.02 ^c^	1.67 ± 0.04 ^d^	<0.0001	<0.0001	<0.0001
Nighttime	1.98 ± 0.05 ^a^	2.96 ± 0.05 ^b^	2.02 ± 0.05 ^a^	3.77 ± 0.06 ^d^	<0.0001	<0.0001	<0.0001
Daily activity (counts)							
Daytime	206 ± 6 ^a^	558 ± 19 ^b^	653 ± 24 ^c^	592 ± 23 ^b^	<0.0001	<0.0001	<0.0001
Nighttime	2829 ± 72 ^a^	1168 ± 34 ^b^	1598 ± 46 ^c^	1313 ± 39 ^b^	<0.0001	<0.0001	<0.0001

Statistical differences were determined using two-way ANOVA examining the effects of maternal obesity and postweaning HF diet. Significant interactions identified by two-way ANOVA were followed by one-way ANOVA and all pair-wise comparisons by Student–Newman–Keuls. ns, no significant interaction. Data are expressed as mean ± SEM. Values with different letters (^a,b,c,d^) are significantly different from each other (*p* < 0.05).

**Table 2:** Analysis of circadian clock gene and appetite gene expression in the offspring Suprachiasmatic Nucleus (SCN) and Arcuate Nucleus (ARC), respectively.

		Offspring of Lean C-fed Dams			Offspring of Obese HF-fed Dams	
		Control (C/C)	HF Diet (C/HF)		Control (HF/C)	HF Diet (HF/HF)
**CLOCK genes**						
***Clock***	Mensor	0.062	0.071		0.064	0.051
	Amplitude	0.016	NSR		NSR	0.017
	Acrophase (ZT:min)	23.41	NSR		NSR	1.93 *
	
***Bmal1***	Mensor	0.012	0.006 *		0.008 *	0.014
	Amplitude	0.012	NSR		NSR	NSR
	Acrophase (ZT:min)	0.39	NSR		NSR	NSR
	
***Per2***	Mensor	0.221	0.084 *		0.161	0.138
	Amplitude	0.089	NSR		0.077	NSR
	Acrophase (ZT:min)	7.01	NSR		10.00	NSR
	
***Cry2***	Mensor	0.401	0.122 *		0.641 *	0.373
	Amplitude	0.150	NSR		0.117	NSR
	Acrophase (ZT:min)	8.34	NSR		2.01 *	NSR
	
**APPETITE genes**						
***Npy***	Mensor	2.654	3.900		2.859	3.348
	Amplitude	1.721	NSR		NSR	NSR
	Acrophase (ZT:min)	17.97	NSR		NSR	NSR
	
***Pomc***	Mensor	1.321	2.084 *		0.936	1.924
	Amplitude	0.897	NSR		NSR	NSR
	Acrophase (ZT:min)	9.27	NSR		NSR	NSR

Cosinor analysis was performed. Offspring group: offspring of lean C-fed dams on C diet (C/C) or HF diet (C/HF), and offspring of obese HF-fed dams on C diet (HF/C) or HF diet (HF/HF). *n* = 6 time points × 6 per time point. * indicates significant differences vs. C/C group (*p* < 0.05) Circadian rhythmicity was considered significant for a *p* < 0.05; NSR, not significantly rhythmic.

## Appendix A

**Table A1:** The dietary composition of macronutrients and energy values of the diets used in this study.

Dietary Composition	Control Diet	High-Fat Diet
Percentage weight:		
Carbohydrate	66.7	39.8
Protein	14.4	23.0
Lipid	2.7	22.6
Percentage energy (kcal):		
Carbohydrate	75.1	35.0
Protein	17.5	20.0
Lipid	7.4	45.0
Energy (AFE MJ/kg)	13.8	19.1
Fat breakdown (% AFE):		
Saturated Fatty Acids (SFA)	0.5	6.5
Monounsaturated Fatty Acids (MUFA)	0.9	6.5
Polyunsaturated Fatty Acids (PUFA)	0.9	4.2

AFE = Atwater Fuel Energy = ((CO%/100) × 9000) + ((CP%/100) × 4000) + ((NFE%/100) × 4000)/239.23.

## References

[1] Shaw J., Sicree R., Zimmet P. (2010). Global estimates of the prevalence of diabetes for 2010 and 2030. Diabetes Res. Clin. Pract..

[2] Nuttall F.Q. (2015). Body Mass Index: Obesity, BMI, and Health: A Critical Review. Nutr. Today.

[3] Kohsaka A., Bass J. (2007). A sense of time: How molecular clocks organize metabolism. Trends Endocrinol. Metab..

[4] Cagampang F.R., Bruce K.D. (2012). The role of the circadian clock system in nutrition and metabolism. Br. J. Nutr..

[5] Lowrey P.L., Takahashi J.S. (2011). Genetics of circadian rhythms in Mammalian model organisms. Genet. Genom. Fish Phenomics.

[6] Dibner C., Schibler U., Albrecht U. (2010). The Mammalian Circadian Timing System: Organization and Coordination of Central and Peripheral Clocks. Annu. Rev. Physiol..

[7] Turek F.W., Joshu C., Kohsaka A., Lin E., Ivanova G., McDearmon E., Laposky A., Losee-Olson S., Easton A., Jensen D.R. (2005). Obesity and metabolic syndrome in circadian Clock mutant mice. Science.

[8] Oishi K., Atsumi G., Sugiyama S., Kodomari I., Kasamatsu M., Machida K., Ishida N. (2006). Disrupted fat absorption attenuates obesity induced by a high-fat diet in Clock mutant mice. FEBS Lett..

[9] Kohsaka A., Laposky A.D., Ramsey K.M., Estrada C., Joshu C., Kobayashi Y., Turek F.W., Bass J. (2007). High-Fat Diet Disrupts Behavioral and Molecular Circadian Rhythms in Mice. Cell Metab..

[10] Challet E. (2013). Circadian Clocks, Food Intake, and Metabolism. Prog. Mol. Biol. Transl. Sci..

[11] Schwartz M.W. (2006). Central Nervous System Regulation of Food Intake. Obesity.

[12] Gehlert D.R., Chronwall B.M., Schafer M.P., O’Donohue T.L. (1987). Localization of neuropeptide Y messenger ribonucleic acid in rat and mouse brain by in situ hybridization. Synapse.

[13] Kageyama H., Takenoya F., Hirako S., Wada N., Kintaka Y., Inoue S., Ota E., Ogawa T., Shioda S. (2012). Neuronal circuits involving neuropeptide Y in hypothalamic arcuate nucleus-mediated feeding regulation. Neuropeptides.

[14] Schwartz M.W., Woods S.C., Porte D., Seeley R.J., Baskin D.G. (2000). Central nervous system control of food intake. Nature.

[15] Atasoy D., Betley J.N., Su H.H., Sternson S.M. (2012). Deconstruction of a neural circuit for hunger. Nature.

[16] Clark J.T., Kalra P.S., Kalra S.P. (1985). Neuropeptide Y Stimulates Feeding but Inhibits Sexual Behavior in Rats. Endocrinology.

[17] Sousa-Ferreira L., Garrido M., Nascimento-Ferreira I., Nobrega C., Santos-Carvalho A., Álvaro A.R., Rosmaninho-Salgado J., Kaster M., Kügler S., De Almeida L.P. (2011). Moderate Long-Term Modulation of Neuropeptide Y in Hypothalamic Arcuate Nucleus Induces Energy Balance Alterations in Adult Rats. PLoS ONE.

[18] Wilding J.P.H., Gilbey S.G., Mannan M., Aslam N., Ghatei M.A., Bloom S.R. (1992). Increased neuropeptide Y content in individual hypothalamic nuclei, but not neuropeptide Y mRNA, in diet-induced obesity in rats. J. Endocrinol..

[19] Dryden S., Pickavance L., Frankish H.M., Williams G. (1995). Increased neuropeptide Y secretion in the hypothalamic paraventricular nucleus of obese (fa/fa) Zucker rats. Brain Res..

[20] Huang X.-F., Han M., Storlien L.H. (2003). The level of NPY receptor mRNA expression in diet-induced obese and resistant mice. Mol. Brain Res..

[21] Souza G.F.P., Solon C., Nascimento L.F., De-Lima-Junior J.C., Nogueira G., Moura R., Rocha G.Z., Fioravante M., Bobbo V., Morari J. (2016). Defective regulation of POMC precedes hypothalamic inflammation in diet-induced obesity. Sci. Rep..

[22] Diane A., Pierce W.D., Russell J.C., Heth C.D., Vine D.F., Richard D., Proctor S.D. (2014). Down-regulation of hypothalamic pro-opiomelanocortin (POMC) expression after weaning is associated with hyperphagia-induced obesity in JCR rats overexpressing neuropeptide Y. Br. J. Nutr..

[23] Xu B., Kalra P.S., Farmerie W.G., Kalra S.P. (1999). Daily Changes in Hypothalamic Gene Expression of Neuropeptide Y, Galanin, Proopiomelanocortin, and Adipocyte Leptin Gene Expression and Secretion: Effects of Food Restriction 1. Endocrinology.

[24] Stütz A.M., Staszkiewicz J., Ptitsyn A., Argyropoulos G. (2007). Circadian Expression of Genes Regulating Food Intake*. Obesity.

[25] Buijs R.M., Hou Y.-X., Shinn S., Renaud L.P. (1994). Ultrastructural evidence for intra- and extranuclear projections of GABAergic neurons of the suprachiasmatic nucleus. J. Comp. Neurol..

[26] Abe M., Herzog E.D., Yamazaki S., Straume M., Tei H., Sakaki Y., Menaker M., Block G.D. (2002). Circadian Rhythms in Isolated Brain Regions. J. Neurosci..

[27] Buijs F.N., Guzman-Ruiz M., Leon-Mercado L., Basualdo M.C., Escobar C., Kalsbeek A., Buijs R.M. (2017). Suprachiasmatic Nucleus Interaction with the Arcuate Nucleus; Essential for Organizing Physiological Rhythms. Eneuro.

[28] Drake A.J., Reynolds R.M. (2010). Impact of maternal obesity on offspring obesity and cardiometabolic disease risk. Reproduction.

[29] Suter M., Bocock P., Showalter L., Hu M., Shope C., McKnight R., Grove K., Lane R., Aagaard-Tillery K. (2011). Epigenomics: Maternal high-fat diet exposure in utero disrupts peripheral circadian gene expression in nonhuman primates. FASEB J..

[30] Borengasser S.J., Kang P., Faske J., Gomez-Acevedo H., Blackburn M.L., Badger T.M., Shankar K. (2014). High fat diet and in utero exposure to maternal obesity disrupts circadian rhythm and leads to metabolic programming of liver in rat offspring. PLoS ONE.

[31] Bruce K.D., Szczepankiewicz D., Sihota K.K., Ravindraanandan M., Thomas H., Lillycrop K.A., Burdge G.C., Hanson M.A., Byrne C.D., Cagampang F.R. (2016). Altered cellular redox status, sirtuin abundance and clock gene expression in a mouse model of developmentally primed NASH. Biochim. Biophys. Acta.

[32] Bruce K.D., Cagampang F.R., Argenton M., Zhang J., Ethirajan P.L., Burdge G.C., Bateman A.C., Clough G.F., Poston L., Hanson M.A. (2009). Maternal high-fat feeding primes steatohepatitis in adult mice offspring, involving mitochondrial dysfunction and altered lipogenesis gene expression. Hepatology.

[33] Elahi M.M., Cagampang F.R., Mukhtar D., Anthony F.W., Ohri S.K., Hanson M.A. (2009). Long-term maternal high-fat feeding from weaning through pregnancy and lactation predisposes offspring to hypertension, raised plasma lipids and fatty liver in mice. Br. J. Nutr..

[34] Sellayah D., Thomas H., Lanham S.A., Cagampang F.R. (2019). Maternal Obesity During Pregnancy and Lactation Influences Offspring Obesogenic Adipogenesis but Not Developmental Adipogenesis in Mice. Nutrients.

[35] Desai M., Jellyman J.K., Han G., Beall M., Lane R.H., Ross M.G., Han G. (2014). Maternal obesity and high-fat diet program offspring metabolic syndrome. Am. J. Obstet. Gynecol..

[36] Samuelsson A.-M., Matthews P.A., Argenton M., Christie M.R., McConnell J.M., Jansen E.H.M., Piersma A.H., Ozanne S.E., Twinn D.F., Remacle C. (2008). Diet-Induced Obesity in Female Mice Leads to Offspring Hyperphagia, Adiposity, Hypertension, and Insulin Resistance: A Novel Murine Model of Developmental Programming. Hypertension.

[37] Ellacott K.L., Morton G.J., Woods S.C., Tso P., Schwartz M.W. (2010). Assessment of feeding behavior in laboratory mice. Cell Metab..

[38] Rajia S., Morris M.J., Chen H. (2010). Maternal overnutrition impacts offspring adiposity and brain appetite markers-modulation by postweaning diet. J. Neuroendocr..

[39] Sclafani A. (1984). Animal models of obesity: Classification and characterization. Int. J. Obes..

[40] Fenton P.F., Carr C.J. (1951). The nutrition of the mouse. XI. Response of four strains to diets differing in fat content. J. Nutr..

[41] Bunger M.K., Wilsbacher L.D., Moran S.M., Clendenin C., Radcliffe L.A., HogenEsch J.B., Simon M.C., Takahashi J.S., Bradfield C.A. (2000). Mop3 Is an Essential Component of the Master Circadian Pacemaker in Mammals. Cell.

[42] Zheng B., Larkin D.W., Albrecht U., Sun Z.S., Sage M., Eichele G., Lee C.C., Bradley A. (1999). The mPer2 gene encodes a functional component of the mammalian circadian clock. Nature.

[43] Bae K., Jin X., Maywood E.S., Hastings M.H., Reppert S.M., Weaver D.R. (2001). Differential functions of mPer1, mPer2, and mPer3 in the SCN circadian clock. Neuron.

[44] Van Der Horst G.T.J., Muijtjens M., Kobayashi K., Takano R., Kanno S.-I., Takao M., De Wit J., Verkerk A., Eker A.P.M., Van Leenen D. (1999). Mammalian Cry1 and Cry2 are essential for maintenance of circadian rhythms. Nature.

[45] Bonaconsa M., Malpeli G., Montaruli A., Carandente F., Grassi-Zucconi G., Bentivoglio M. (2014). Differential modulation of clock gene expression in the suprachiasmatic nucleus, liver and heart of aged mice. Exp. Gerontol..

[46] Akabayashi A., Levin N., Paez X., Alexander J.T., Leibowitz S.F. (1994). Hypothalamic Neuropeptide Y and Its Gene Expression: Relation to Light/Dark Cycle and Circulating Corticosterone. Mol. Cell. Neurosci..

[47] Steiner R.A., Kabigting E., Lent K., Clifton D.K. (1994). Diurnal Rhythm in Proopiomelanocortin mRNA in the Arcuate Nucleus of the Male Rat. J. Neuroendocr..

[48] Guerrero-Vargas N.N., Espitia-Bautista E., Buijs R.M., Escobar C. (2018). Shift-work: Is time of eating determining metabolic health? Evidence from animal models. Proc. Nutr. Soc..

[49] Espitia-Bautista E., Velasco-Ramos M., Osnaya-Ramírez I., Ángeles-Castellanos M., Buijs R.M., Escobar C. (2017). Social jet-lag potentiates obesity and metabolic syndrome when combined with cafeteria diet in rats. Metabolism.

[50] Brum M.C.B., Filho F.F.D., Schnorr C.C., Bottega G.B., Rodrigues T.C. (2015). Shift work and its association with metabolic disorders. Diabetol. Metab. Syndr..

[51] Fonken L.K., Workman J.L., Walton J.C., Weil Z.M., Morris J.S., Haim A., Nelson R.J. (2010). Light at night increases body mass by shifting the time of food intake. Proc. Natl. Acad. Sci. USA.

[52] Pietroiusti A., Neri A., Somma G., Coppeta L., Iavicoli I., Bergamaschi A., Magrini A. (2010). Incidence of metabolic syndrome among night-shift healthcare workers. Occup. Environ. Med..

[53] Oosterman J.E., Kalsbeek A., la Fleur S.E., Belsham D.D. (2015). Impact of nutrients on circadian rhythmicity. Am. J. Physiol. Regul. Integr. Comp. Physiol..

[54] Pendergast J.S., Branecky K.L., Yang W., Ellacott K.L., Niswender K.D., Yamazaki S. (2013). High-fat diet acutely affects circadian organisation and eating behavior. Eur. J. Neurosci..

[55] Krämer A., Yang F.-C., Snodgrass P., Li X., Scammell T.E., Davis F.C., Weitz C.J. (2001). Regulation of Daily Locomotor Activity and Sleep by Hypothalamic EGF Receptor Signaling. Science.

[56] Jordan L.M. (1998). Initiation of locomotion in mammals. Ann. N. Y. Acad. Sci..

[57] Ibrahim N., Bosch M.A., Smart J.L., Qiu J., Rubinstein M., Rønnekleiv O.K., Low M.J., Kelly M.J. (2003). Hypothalamic Proopiomelanocortin Neurons Are Glucose Responsive and Express K ATP Channels. Endocrinology.

[58] Thaler J.P., Yi C.-X., Schur E.A., Guyenet S.J., Hwang B.H., Dietrich M.O., Zhao X., Sarruf D.A., Izgur V., Maravilla K.R. (2012). Obesity is associated with hypothalamic injury in rodents and humans. J. Clin. Investig..

[59] Gropp E., Shanabrough M., Borok E., Xu A.W., Janoschek R., Buch T., Plum L., Balthasar N., Hampel B., Waisman A. (2005). Agouti-related peptide–expressing neurons are mandatory for feeding. Nat. Neurosci..

[60] Dalvi P.S., Chalmers J.A., Luo V., Han D.Y., Wellhauser L., Liu Y., Tran D.Q., Castel J., Luquet S., Wheeler M.B. (2017). High fat induces acute and chronic inflammation in the hypothalamus: Effect of high-fat diet, palmitate and TNF-alpha on appetite-regulating NPY neurons. Int. J. Obes..

[61] Roseberry A.G., Liu H., Jackson A.C., Cai X., Friedman J.M. (2004). Neuropeptide Y-mediated inhibition of proopiomelanocortin neurons in the arcuate nucleus shows enhanced desensitization in ob/ob mice. Neuron.

[62] Klein M.O., Mackay H., Edwards A., Park S.-B., Kiss A.C.I., Felicio L.F., Abizaid A. (2018). POMC and NPY mRNA expression during development is increased in rat offspring brain from mothers fed with a high fat diet. Int. J. Dev. Neurosci..

[63] Kojima S., Catavero C., Rinaman L. (2016). Maternal high-fat diet increases independent feeding in pre-weanling rat pups. Physiol. Behav..

[64] Harrold J.A., Dovey T.M., Blundell J.E., Halford J.C. (2012). CNS regulation of appetite. Neuropharmacology.

[65] Amin T., Mercer J.G. (2016). Hunger and Satiety Mechanisms and Their Potential Exploitation in the Regulation of Food Intake. Curr. Obes. Rep..

[66] Tremblay A., Bellisle F. (2015). Nutrients, satiety, and control of energy intake. Appl. Physiol. Nutr. Metab..

[67] Ong Z.Y., Muhlhausler B.S. (2011). Maternal “junk-food” feeding of rat dams alters food choices and development of the mesolimbic reward pathway in the offspring. FASEB J..

[68] Nascimento L.F., Souza G.F., Morari J., Barbosa G.O., Solon C., Moura R.F., Victorio S.C., Ignacio-Souza L.M., Razolli D.S., Carvalho H.F. (2016). n-3 Fatty Acids Induce Neurogenesis of Predominantly POMC-Expressing Cells in the Hypothalamus. Diabetes.

[69] Benzler M., Benzler J., Stoehr S., Hempp C., Rizwan M.Z., Heyward P., Tups A. (2019). “Insulin-like” effects of palmitate compromise insulin signalling in hypothalamic neurons. J. Comp. Physiol. B.

[70] Kuljis D.A., Loh D.H., Truong D., Vosko A.M., Ong M.L., McClusky R., Arnold A.P., Colwell C.S. (2013). Gonadal- and sex-chromosome-dependent sex differences in the circadian system. Endocrinology.

[71] Aagaard-Tillery K.M., Grove K., Bishop J., Ke X., Fu Q., McKnight R., Lane R.H. (2008). Developmental origins of disease and determinants of chromatin structure: Maternal diet modifies the primate fetal epigenome. J. Mol. Endocrinol..

[72] Keleher M.R., Zaidi R., Shah S., Oakley M.E., Pavlatos C., El Idrissi S., Xing X., Li D., Wang T., Cheverud J.M. (2018). Maternal high-fat diet associated with altered gene expression, DNA methylation, and obesity risk in mouse offspring. PLoS ONE.

[73] Ramamoorthy T.G., Allen T.-J., Davies A., Harno E., Sefton C., Murgatroyd C., White A. (2018). Maternal overnutrition programs epigenetic changes in the regulatory regions of hypothalamic Pomc in the offspring of rats. Int. J. Obes..

[74] Ramos-Lopez O., Samblas M., Milagro F.I., Riezu-Boj J.I., Crujeiras A., Martinez J.A., Project M. (2018). Circadian gene methylation profiles are associated with obesity, metabolic disturbances and carbohydrate intake. Chronobiol. Int..

[75] Ji Y., Qin Y., Shu H., Li X. (2010). Methylation analyses on promoters of mPer1, mPer2, and mCry1 during perinatal development. Biochem. Biophys. Res. Commun..

[76] Doi M., Hirayama J., Sassone-Corsi P. (2006). Circadian Regulator CLOCK Is a Histone Acetyltransferase. Cell.

[77] Sridhar S.B., Darbinian J., Ehrlich S.F., Markman M.A., Gunderson E.P., Ferrara A., Hedderson M.M. (2014). Maternal gestational weight gain and offspring risk for childhood overweight or obesity. Am. J. Obstet. Gynecol..

[78] Parlee S.D., MacDougald O.A. (2014). Maternal nutrition and risk of obesity in offspring: The Trojan horse of developmental plasticity. Biochim. Biophys. Acta.

[79] Bruce K.D., Cagampang F.R., Argenton M., Zhang J.L., Ethirajan P., Clough G.F., Poston L., Hanson M.A., McConnell J., Byrne C.D. (2009). Maternal Exposure to a High Fat Diet Increases Susceptibility to Adult Onset of Non-Alcoholic Fatty Liver Disease (NAFLD). Reprod. Sci..

[80] Zhang J., Zhang F., Didelot X., Bruce K.D., Cagampang F.R., Vatish M., Hanson M., Lehnert H., Ceriello A., Byrne C.D. (2009). Maternal high fat diet during pregnancy and lactation alters hepatic expression of insulin like growth factor-2 and key microRNAs in the adult offspring. BMC Genom..

[81] Chong N.W.S., Cagampang F.R.A., Coen C.W., Campbell I.C., Powell J.F. (1996). Rapid identification of novel genes expressed in a circadian manner in rat suprachiasmatic nuclei. NeuroReport.

[82] Cleal J.K., Shepherd J.N., Shearer J.L., Bruce K.D., Cagampang F.R. (2014). Sensitivity of housekeeping genes in the suprachiasmatic nucleus of the mouse brain to diet and the daily light–dark cycle. Brain Res..

